# Distribution of airway pressure opening in the lungs measured with electrical impedance tomography (POET): a prospective physiological study

**DOI:** 10.1186/s13054-025-05264-3

**Published:** 2025-01-16

**Authors:** Nannan Sun, Clement Brault, Antenor Rodrigues, Matthew Ko, Fernando Vieira, Vorakamol Phoophiboon, Michel Slama, Lu Chen, Laurent Brochard

**Affiliations:** 1 Keenan Centre for Biomedical Research, Li Ka Shing Knowledge Institute, Unity Health Toronto, Toronto, ON Canada; 2 Interdepartmental Division of Critical Care Medicine, University of Toronto, Toronto, ON Canada; 3 Department of Critical Care Medicine and Anesthesia Intensive Care Unit, The First Affiliated Hospital of Shandong First Medical University & Shandong Provincial Qianfoshan Hospital, Shandong Institute of Anesthesia and Respiratory Critical Medicine, Shandong Provincial Clinical Research Center for Anesthesiology, Jinan, Shandong China; 4 Intensive Care Department, Amiens-Picardie University Hospital, Amiens, France; 5 Division of Critical Care Medicine, Department of Medicine, Faculty of Medicine, Chulalongkorn University, Bangkok, Thailand

**Keywords:** Acute hypoxemic respiratory failure, Mechanical ventilation, Electrical impedance tomography, Airway opening pressure, Low-flow insufflation maneuver, Low-flow pressure–time curve

## Abstract

**Background:**

In patients with acute hypoxemic respiratory failure (AHRF) under mechanical ventilation, the change in pressure slope during a low-flow insufflation indicates a global airway opening pressure (AOP) needed to reopen closed airways and may be used for titration of positive end-expiratory pressure.

**Objectives:**

To understand 1) if airways open homogeneously inside the lungs or significant regional AOP variations exist; 2) whether the pattern of the pressure slope change during low-flow insufflation can indicate the presence of regional AOP variations.

**Methods:**

Using electrical impedance tomography, we recorded low-flow insufflation maneuvers (< 10 L/min) starting from end-expiratory positive pressure 0–5 cmH_2_O. We measured global (AOP_global_) and regional AOPs from pressure-impedance curves in the four different lung quadrants, and compared AOP_global_ with the highest quadrantal AOP (AOP_highest_). We categorized the slope change of the low-flow inflation pressure–time curve into three patterns: no change, progressive change, abrupt change.

**Results:**

Among the 36 patients analyzed, 9 (25%) had AOP_global_ ≥ 5 cmH_2_O whereas 19 (53%) exhibited regional AOP_highest_ ≥ 5 cmH_2_O. AOP_global_ was on average similar to AOP of the upper right quadrant (*P* = 0.182) but was lower than AOPs of the other three quadrants (*P* < 0.01 of each). AOP_global_ was significantly lower than AOP_highest_: 3.0 [2.0–4.3] *vs.* 5.0 [2.8–8.3] cmH_2_O, *P* < 0.001. AOP was higher in the dependent than the non-dependent ventilated lung (4.0 [2.0–6.3] *vs*. 3.0 [2.0–5.0] cmH_2_O, *P* < 0.001). Seventeen (47%) patients exhibited a ‘progressive change’ pattern in the pressure–time curve. These patients had a larger difference between AOP_highest_ and AOP_global_ (3.0 [2.0–4.0] cmH_2_O with a maximum of 8 cmH_2_O) compared to the other two patterns: 1.0 [0–1.0] cmH_2_O in ‘no change’ , *P* < 0.001 and 1.0 [0–2.0] cmH_2_O in ‘abrupt change’ , *P* = 0.003.

**Conclusion:**

AOP_global_ mostly reflects the lowest opening pressure in the lung and frequently underestimates the highest regional AOP in mechanically ventilated patients with AHRF. A progressive slope change during the low-flow pressure–time curve indicates the presence of several and higher regional AOPs.

**Trial registration:**

Clinicaltrials.gov, NCT 05825534 (registered, April 24th, 2023), retrospectively registered.

**Graphical abstract:**

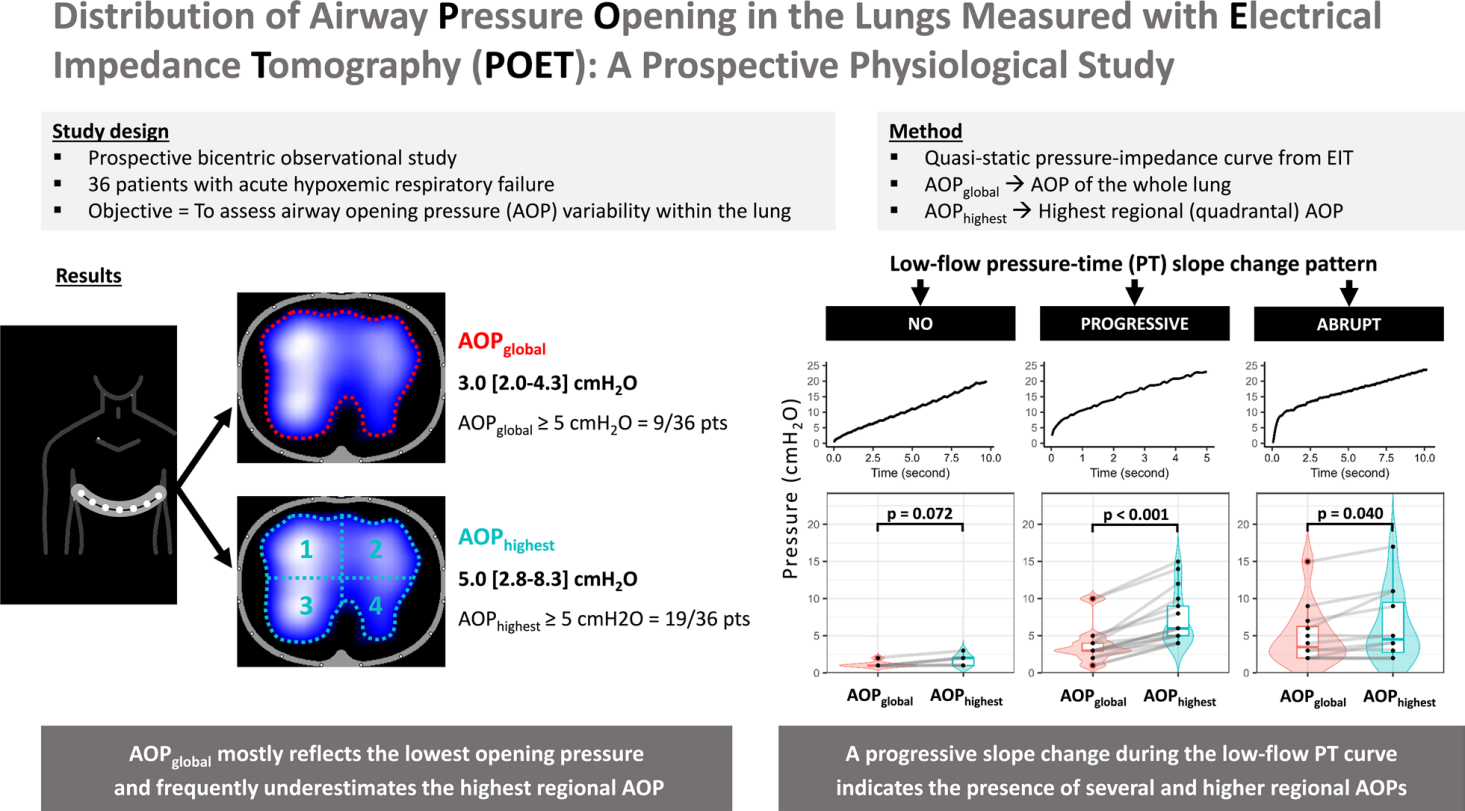

**Supplementary Information:**

The online version contains supplementary material available at 10.1186/s13054-025-05264-3.

## Background

Complete airway closure affects approximately 30% of patients with acute hypoxemic respiratory failure (AHRF) [1]. In these patients, airway collapse occurs during expiration, when the airway pressure (Paw) is reduced. This phenomenon differs from atelectasis, where alveoli are collapsed, since alveoli remain aerated in the case of airway closure [2]. During insufflation, lung inflation starts only when the Paw is above a level of airway opening pressure (AOP), corresponding to the pressure to overcome airway closure [3]. Although the level of AOP can be low it can be up to 20 cmH_2_O in some patients [4].

The standard method for detecting complete airway closure and measuring AOP is based on a simple quasi-static pressure–volume (PV) or pressure–time (PT) curve obtained during a low-flow insufflation maneuver, typically starting from a positive end-expiratory pressure (PEEP) of 0–5 cmH_2_O [3]. This simple bedside method reflects the behavior of the lung (as a mono-compartmental model). However, AHRF, especially acute respiratory distress syndrome (ARDS), is characterized by a high degree of lung heterogeneity, leading to a different distribution of the tidal ventilation, and potential variations of regional airway closure and regional AOP within the lung [2, 5]. As the global AOP level could serve as a logical reference to set a minimal PEEP, it is worthwhile to assess whether and how regional AOPs could be higher than the global AOP.

Electrical impedance tomography (EIT) is a non-invasive and radiation-free imaging technique which measures the regional change in lung impedance caused by the change in air content. It provides valuable information for setting mechanical ventilation, such as PEEP selection [6]. Rozé et al. recorded impedance signals during low-flow insufflation maneuvers and reconstructed regional quasi-static pressure-impedance curves to detect regional airway closure in patients with asymmetrical ARDS [5, 7]. They found a substantial difference between AOP levels of each of the two lungs: the more injured lung, the higher regional AOP. However, the global AOP measured from the quasi-static PV curve was more indicating the AOP of the less injured lung and therefore systematically underestimated the AOP of the sickest lung.

Based on these findings, the quasi-static PV curve from the ventilator may represent the minimal AOP and may not accurately reflect regional AOPs. In this study, we used quasi-static pressure-impedance curves from EIT to assess the prevalence and distribution of regional airway closure and to measure the level of regional AOP within the lung of patients with AHRF.

## Methods

### Patient enrollment

**Fig. 1:**
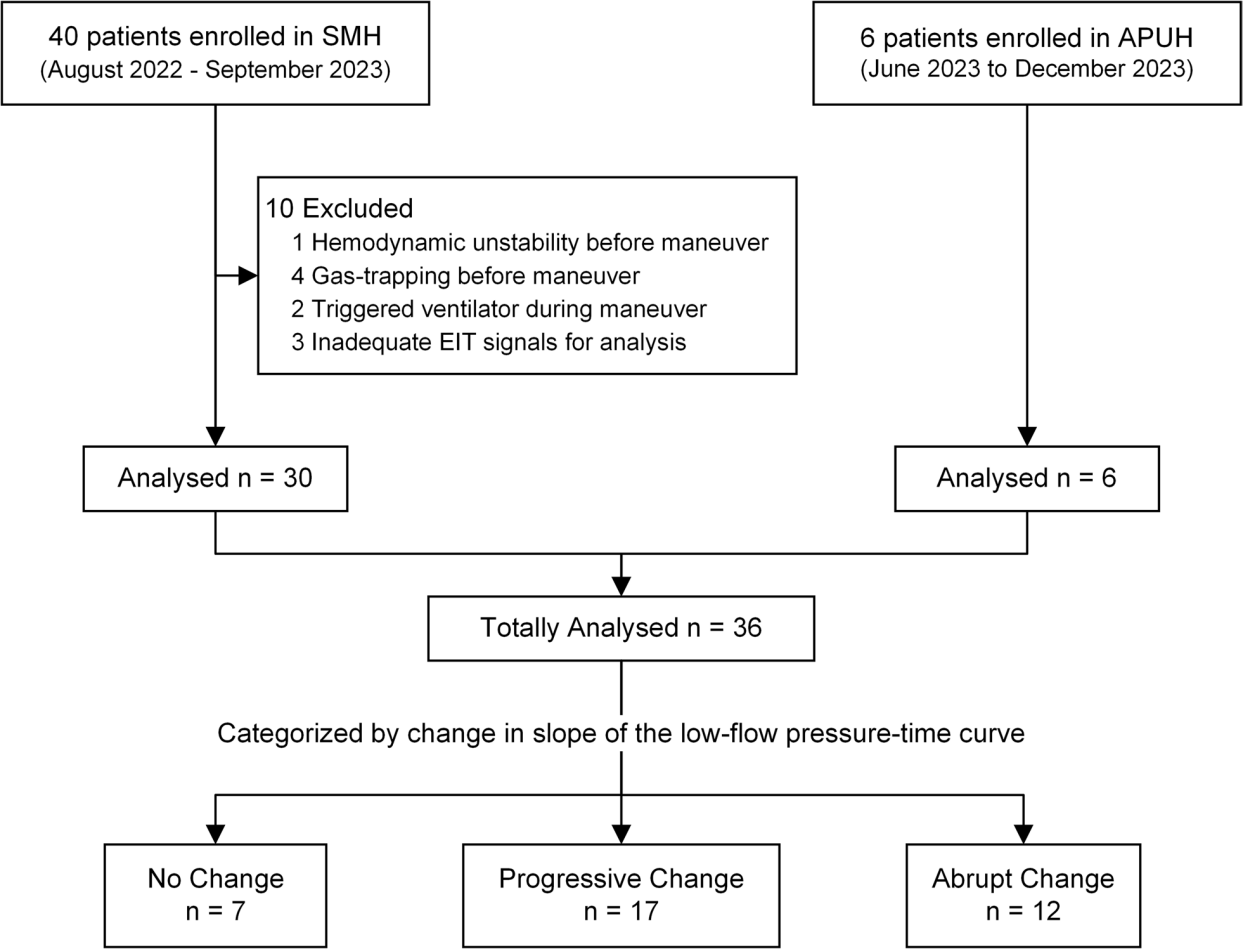
Flowchart of POET study

This prospective bicentric observational study was approved by the Unity Health Toronto Research Ethics Board (REB#22–079) and the appropriate French research ethics committee (Comité de Protection des Personnes Est III, Ref#2022-A02811-42), registered (NCT05825534), and conducted in two intensive care units (ICUs) at St. Michael’s Hospital, Unity Health, (SMH, Toronto, Canada) and Amiens-Picardie University Hospital (APUH, Amiens, France). Regardless of the etiology, passively mechanically ventilated patients in the ICUs were screened for enrollment. We enrolled adult patients (aged ≥ 18 years) with AHRF or ARDS, who were deeply sedated and/or paralyzed as per clinical decision with a ratio of partial pressure of oxygen in arterial blood to fraction of inspired oxygen (P_a_O_2_/FiO_2_) ≤ 300 mmHg. Exclusion criteria included pneumothorax and bronchopleural fistula, severe hypoxemia (P_a_O_2_/FiO_2_ ratio < 80 mmHg), and severe hemodynamic instability (> 30% increase in vasopressors in the last 6 h or norepinephrine > 0.5 µg/kg/min). For a complete list, please see previous reference [8]. Informed or deferred consent was obtained from each patient’s legal substitute decision-maker before the onset of any study procedures (Flowchart see Fig. 1).

### Study protocol

#### Device installation and settings

Before the maneuver, we ensured that patients were passively ventilated (i.e., without spontaneous effort). Patients were ventilated in a standardized volume or pressure control mode using Evita XL or V500 (Dräger, Lübeck, Germany), Servo-i (MAQUET, Getinge, Sweden), or Puritan Bennett (PB) 840 (Medtronic, Boulder, CO, USA). Ventilator settings were at the discretion of the clinical team. At enrollment, we collected demographic and clinical data for each patient.

Airway pressure and flow signals were captured using an analog-to-digital converter (MP150, Biopac systems, Goleta, CA, USA), which was equipped with a differential pressure transducer (TSD160A, Biopac systems, Goleta, CA, USA) and a pneumotachograph (Fleisch A, Lausanne, Switzerland) both at a high sampling frequency of 1000 Hz positioned at the Y-piece. Alternatively, a dedicated system (Fluxmed, MBMed, Martinez, Argentina) was utilized at a sampling rate of 256 Hz per acquisition channel. Pressure and flow transducers underwent calibration before measurements, and volumes were calculated by integrating flow signals. Careful measures were taken to eliminate potential gas leaks, such as removing end-tidal capnography (side-stream sampling air).

EIT was performed using PulmoVista 500 (Dräger, Lübeck, Germany) with a 16-electrode belt placed between the 4th and 5th intercostal space, while synchronously collecting impedance tracings and Paw from PressurePod of the EIT device at 50 Hz. Low-pass filter was set at a cut-off frequency of 50/min.

#### Low-flow insufflation maneuver

We performed low-flow (i.e., < 10 L/min) insufflation maneuvers starting from PEEP of 0 cmH_2_O (n = 35) for most patients or 5 cmH_2_O (n = 1) in one patient with severe and persistent hypoxemia. We limited the maximal Paw to the plateau pressure achieved within clinical ventilator settings and adjusted FiO_2_ based on patient tolerance. Before the maneuver, we transitory reduced respiratory rate (RR) to extend expiration phase and minimize auto-PEEP. Of note, for Evita XL and V500, we used the special ‘low-flow PV loop’ function but we had first to turn down the baseline PEEP, drop RR to 5/min, wait for 2 breaths, and then start the maneuver. For Servo-i and PB840, we recorded 2 low-flow insufflation maneuvers after dropping RR at 5/min or 3/min. We noticed that the exhalation time before the first maneuver was not always long enough to eliminate any auto-PEEP, and decided to keep the second maneuver only.

### Analysis

All recorded signals were analyzed offline using AcqKnowledge software (v4.2, Biopac Systems, Goleta, CA, USA) and EIT Data Analysis Tool (PV500DataAnalysis130, Dräger, Lübeck, Germany). Impedance signals were synchronized with Paw and flow measurements resampled to 50 Hz. All of the data visualization analyses were conducted in R version 4.3.1. For further information see methodological aspects in Additional file 1.

#### Quasi-static pressure–volume curve

**Fig. 2:**
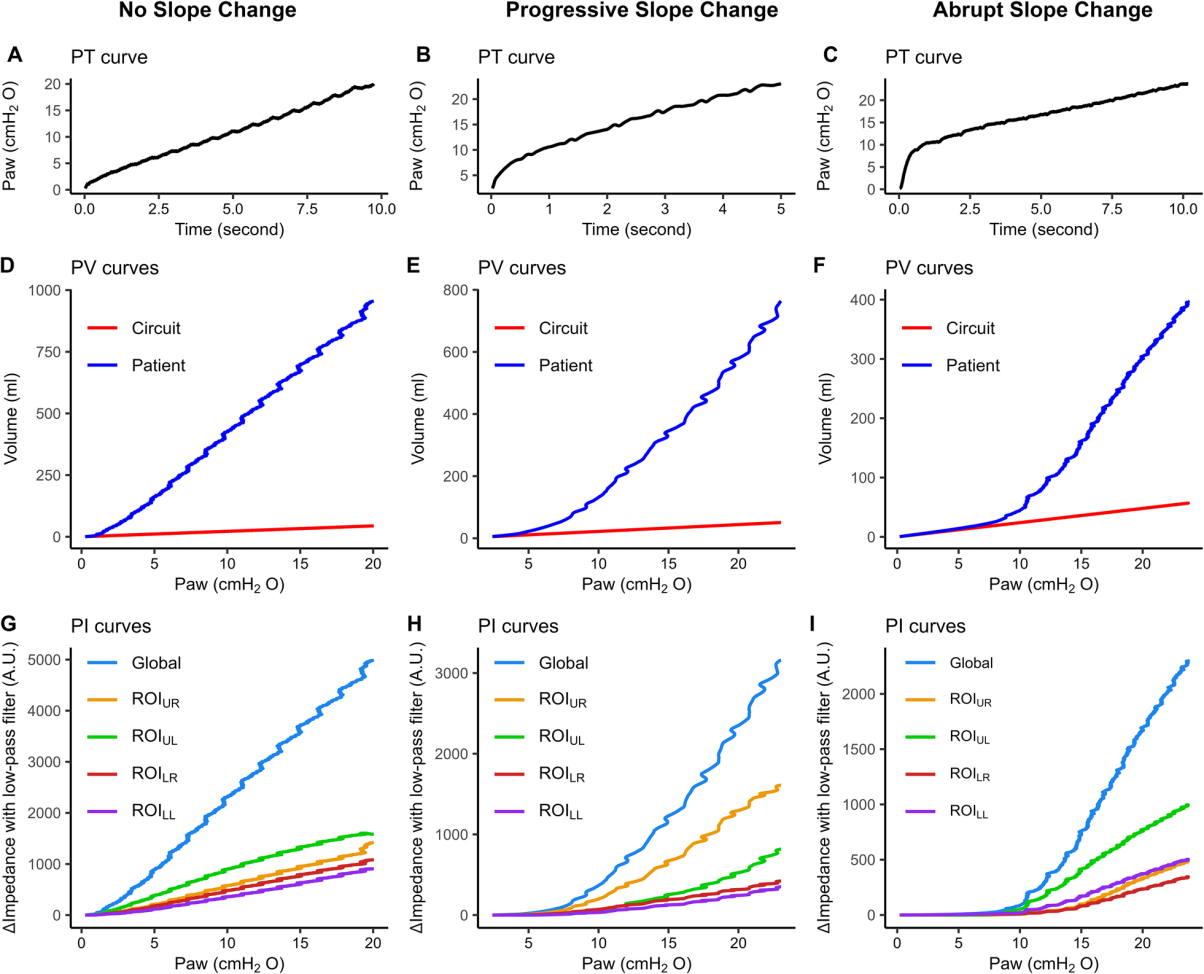
Categorization of change in slope of the pressure–time curve during the low-flow insufflation. Paw-time (PT) curves (**A**–**C**) in different groups. Pressure–volume (PV) curves (**D**–**F**) obtained from patients (in blue) and measured in the bench model with an occluded ventilator circuit (in red). Pressure-impedance (PI) curves (**G**–**I**) obtained from patients in global (in light blue) and quadrant regions: upper right lung (ROI_UR_) in orange, upper left lung (ROI_UL_) in green, lower right lung (ROI_LR_) in dark red, and lower left lung (ROI_LL_) in purple. Panels **A**, **D**, and **G** are typical curves of a no slope change pattern (patient #15). Panels **B**, **E**, and **H** are typical curves of a progressive slope change pattern (patient #35). Panels **C**, **F** and **I** are typical curves of an abrupt slope change pattern (patient #14)

From the quasi-static PV curve, we detected complete airway closure by measuring the AOP as described by Chen et al. [3] (Fig. 2). AOP measured from the quasi-static PV curve (AOP_vent_) was defined as the Paw (round up to integer) at which gas volume delivered to a patient becomes 15 ml greater than the volume compressed in the occluded circuit [9].

#### Quasi-static pressure-impedance curve

EIT images were segmented into four regions of interest (ROIs) representing the upper right (ROI_UR_), upper left (ROI_UL_), lower right (ROI_LR_), and lower left (ROI_LL_) quadrant of the lung (Additional file 1: Fig. S1). The horizontal center of the ventilated lung was defined according to the ventilated contour (i.e. regions where ventilation-related impedance changes occur, same as the functional EIT ventilation images [10]) rather than the thoracic contour, to avoid having dependent ROIs with no or very little ventilation. In each ROI, we measured the regional AOP as the start of insufflation in this ROI. Because the baseline impedance signal fluctuated, we used the following procedure to determine this moment.

Any regional AOP could not be lower than AOP_vent_, derived from the PV curve as a comparative benchmark. Then, we identified the cardiac-induced noise on impedance signal (with a low-pass filter). Immediately before the maneuver, we established a stable baseline when the flow was close to 0 L/min to ensure minimal gas movement. This baseline’s duration was long enough to encompass one complete heartbeat cycle. We determined the upper impedance limit (Fig. S4) as mean plus 2 standard deviations of impedance signal during this baseline. Last, from the quasi-static pressure-impedance curve during the maneuver (Fig. 2) we measured regional AOP, defined as the Paw (round up to integer) at which the unfiltered impedance value (Fig. S3) became greater than the upper impedance limit, indicating the onset of lung inflation in that region. Our approach allowed us to measure AOPs in regions having very different percentages of total ventilation.

This procedure was repeated in the non-dependent and dependent ventilated lung, the left and right lung, as well as the global AOP measured by EIT (AOP_global_). We determined the highest regional AOP measured among the four quadrants (AOP_highest_). Airway closure was considered “clinically relevant” when AOP ≥ 5 cmH_2_O.

Furthermore, we calculated the regional respiratory system compliance (Crs) as the ratio between regional tidal volume (based on the percentage of ventilation in the specific ROI measured during tidal ventilation at clinical PEEP) and the driving pressure corrected to corresponding regional AOP. This calculation assumes that the driving pressure is equally distributed in the entire lung. In cases where the regional AOP exceeded clinical total PEEP, the driving pressure was calculated as the difference between the plateau pressure and the regional AOP, as previously described [5]. Conversely, when a regional AOP was lower than total PEEP, the driving pressure was determined as the difference between the plateau pressure and total PEEP. We also calculated the normalized elastance (elastance normalized to predicted body weight).

#### Aspect of the quasi-static pressure–time curve

The shape of the PT curve during continuous low-flow insufflation can identify global AOP by an abrupt transition from a steep to a lower slope in instances of complete airway closure [4]. The point of inflection of the PT curve indicates the AOP. We categorized the change in slope of the quasi-static PT curve into three patterns: no change (a linear increase in Paw over time), a progressive change (a gradual change in convexity, without a single marked inflection point), and an abrupt change (with marked inflection point) (Fig. 2A–C). The change in slope was assessed by a careful visual inspection of the curve by three independent investigators. Disagreements occurred in 13 of 36 cases (36%), and each discrepancy was discussed until a consensus was reached.

### Statistical analysis

A detailed description of the sample size calculation and post-hoc power analysis was provided in Supplementary Materials. Data are expressed as median (interquartile range). Statistical comparisons between or among AOPs were conducted using paired Wilcoxon or Friedman tests, with Kruskal test (Dunn test with p-adjustment: Holm) applied for comparisons among groups. Spearman test was used for correlation analysis. Dichotomous or nominal categorical variables were statistically compared with Chi-square test. Subgroup analysis includes Berlin ARDS criteria, asymmetrical lung involvement (difference between left and right ventilation > 20%), and obesity (body mass index [BMI] ≥ 30 kg/m^2^). All Statistical tests were two-tailed, and a *p*-value < 0.05 was considered significant. Statistical analyses were conducted in R version 4.3.1.

## Results

### Patients characteristics

Thirty-six patients (median age: 57 [44–72] years, 23 (64%) male) were analyzed (Table 1). The average P_a_O_2_/FiO_2_ was 189 [146–240] mmHg, with PEEP 10 [10–12] cmH_2_O, and Crs 32.9 [26.2–41.7] ml/cmH_2_O. Thirty-two patients (89%) were diagnosed with ARDS and 11 patients (31%) had obesity.

**Table 1:** Characteristics of the patients

Characteristics	Overall (n = 36)	Change in slope of the quasi-static pressure–time curve			
		No Change (n = 7)	Progressive Change (n = 17)	Abrupt Change (n = 12)	*P* value
Males, n(%)	23(64)	7(100)	11(65)	5(42)	0.038
Age, yr	57[44–72]	52[39–74]	58[48–74]	59[47–69]	0.928
Height, cm	172[162–180]	182[178–185]	172[160–175]^a^	168[161–176]^a^	0.017
Body mass index, kg/m^2^	26.0[23.7–31.6]	24.2[20.8–24.6]	26.6[24.8–30.8]	27.9[22.9–32.2]	0.216
Obesity, n(%)	11(31)	1(14)	5(29)	5(42)	0.453
APACHE II at admission	27[21–33]	20[19–29]	28[26–35]	27[25–28]	0.428
*Etiology*					
Pneumonia, n(%)	11(31)	3(43)	6(35)	2(17)	0.663
Aspiration, n(%)	8(22)	2(29)	3(18)	3(25)	
Trauma, n(%)	5(14)	1(14)	2(12)	2(17)	
Extrapulmonary sepsis, n(%)	5(14)	0(0)	4(23)	1(8)	
Others, n(%)	7(19)	1(14)	2(12)	4(33)	
*ARDS, n(%)*	32(89)	6(86)	16(94)	10(83)	0.632
Mild, n(%)	14(44)	3(50)	6(37)	5(50)	0.501
Moderate, n(%)	15(47)	3(50)	7(44)	5(50)	
Severe, n(%)	3(9)	0(0)	3(19)	0(0)	
*At Enrollment*					
SOFA	10[9–13]	9[8–11]	11[10–13]	10[10–14]	0.287
Previous duration of ventilation, days	2[1–4]	1[0.5–2]	3[1–4]	2[1–3]	0.216
P_a_O_2_/FiO_2_ at clinical PEEP, mmHg	189[146–240]	237[167–254]	180[146–218]	186[153–258]	0.446
Clinical PEEP, cmH_2_O	10[10–12]	8[5–9]	12[10–12]^b^	10[10, 11]	0.002
Total PEEP, cmH_2_O	11[10–12]	8[6–10]	12[11–13]^b^	10[10–12]	0.003
Tidal volume, ml	380[332–422]	450[440–480]	360[300–410]^b^	370[332–412]^b^	0.002
Plateau pressure, cmH_2_O	23[19–27]	16[15–20]	27[21–28]^b^	23[21–24]^a^	0.006
Driving pressure, cmH_2_O	11[9–15]	8[8–10]	12[10–16]	10[11–14]	0.068
Corrected driving pressure, cmH_2_O	11[9–15]	8[8–10]	12[10–16]	10[11–14]	0.069
Corrected Crs at clinical PEEP, ml/cmH_2_O	32.9[26.6–41.7]	52.5[50.0–56.2]	30.0[19.4–37.3]^b^	34.2[27.2–38.0]^a^	0.008
Corrected Normalized Elastance, cmH_2_O/ml/kg PBW	1.76[1.49–2.64]	1.34[1Phillips.33–1.59]	2.47[1.56–2.68]	1.74[1.62–2.33]	0.082
AOP_vent_, cmH_2_O	3.0 [1.5–4.0]	1.0[1.0–1.0]	3.0[3.0–4.0]^b^	3.0[2.0–6.0]^b^	0.001

Dichotomous or nominal categorical variables are described in numbers (percentage); continuous variables are described as median [interquartile ranges], as appropriate. Chi-square test (Gender, ARDS) & Kruskal–Wallis H test, Dunn test (p.adjust Holm)

^a^*P* < 0.05 compared to ‘No Slope Change’ group, ^b^*P* < 0.01 compared to ‘No Slope Change’ group

### Global vs. highest regional airway opening pressure

**Fig. 3:**
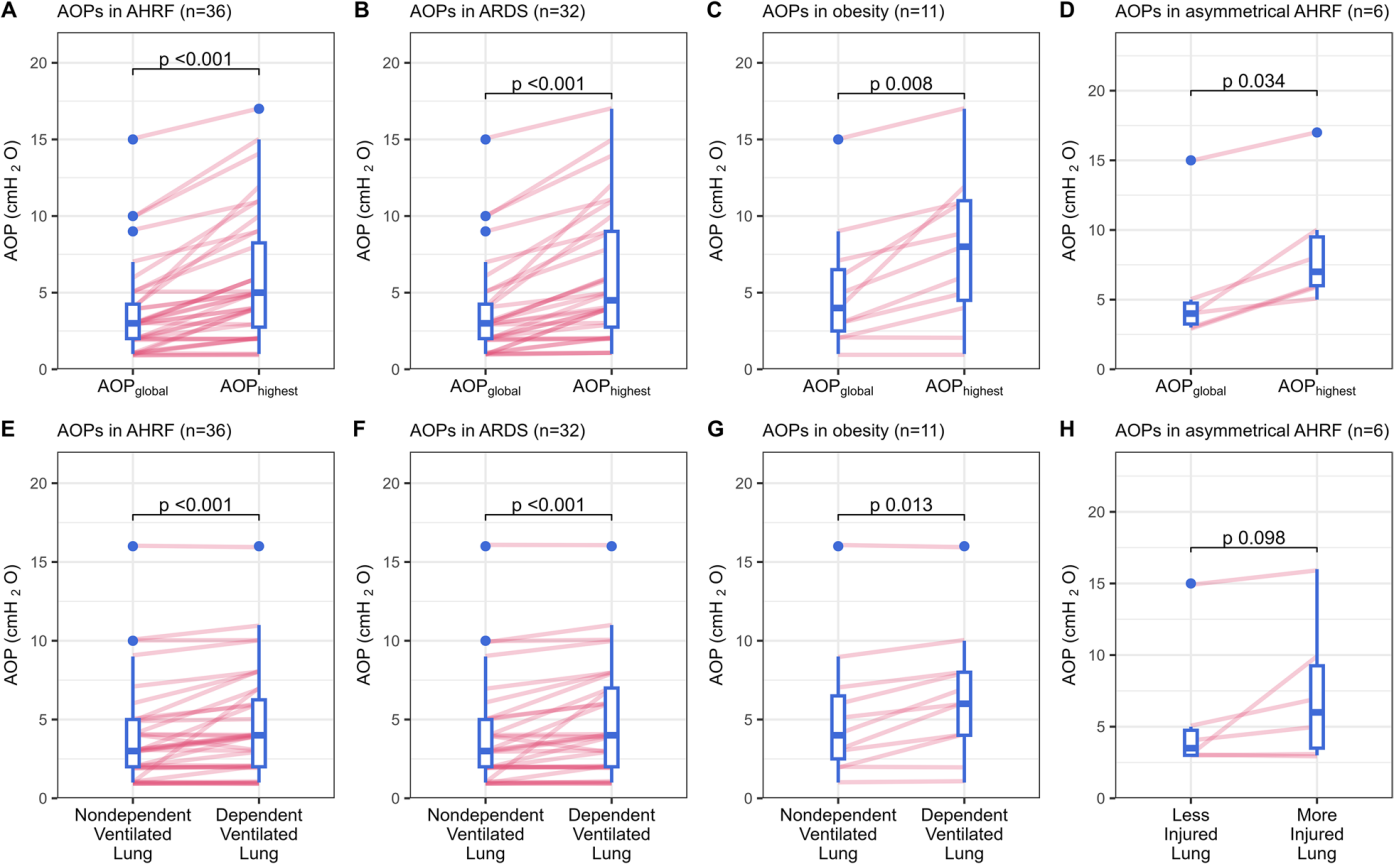
Comparison of global and regional AOPs. Panels **A** and **E** show comparison in all patients. subgroups comparison are ARDS (Panel **B**, **F**), Obesity (Panel **C**, **G**), and Asymmetrical AHRF (Panel **D**, **H**)

The median AOP_global_ (from quasi-static pressure-impedance curve) was 3.0 [2.0–4.3] cmH_2_O, and similar to AOP_vent_ (from quasi-static PV curve) (Additional file 1: Fig. S2). AOP_global_ ranged from 1 to 15 cmH_2_O and was lower than AOP_highest_ that ranged from 1 to 17 cmH_2_O (3.0 [2.0–4.3] vs. 5.0 [2.8–8.3] cmH_2_O, *P* < 0.001) (Fig. 3A). Nine (25%) patients had AOP_global_ ≥ 5 cmH_2_O while 19 (53%) patients exhibited AOP_highest_ ≥ 5 cmH_2_O. More details concerning the prevalence and locations are provided in Additional file 1: Tables S1 and S2.

### Distribution of regional airway opening pressures

AOP_global_ was similar to AOP of the upper right quadrant (*P* = 0.182) but was lower than AOPs of the other three quadrants (*P* < 0.01 of each). Twenty out of 36 patients had a high regional AOP with a difference of AOP_highest_ and AOP_global_ ≥ 2 cmH_2_O, and half of them had more than one quadrant with higher regional AOP within the lung (Additional file 1: Tables S3, S4).

AOP was higher in the dependent ventilated lung than in the non-dependent and AOP_global_ (4.0 [2.0–6.3] *vs*. 3.0 [2.0–5.0] cmH_2_O *vs*. 3.0 [2.0–4.3] cmH_2_O, *P* < 0.001 respectively) (Fig. 3E). AOP of the left lung was slightly higher than the right lung (3.0 [2.0–7.0] *vs*. 3.0 [2.0–5.0] cmH_2_O, *P* = 0.009) and than AOP_global_ (3.0 [2.0–4.3] cmH_2_O, *P* = 0.002).

### Effect of AOP on compliance and normalized elastance

Two patients (6%) had an AOP_global_ (as well as each regional AOP) higher than or equal to total PEEP. Two additional patients had AOP_global_ lower than total PEEP but 1 quadrantal regional AOP exceeded it by 2 cmH_2_O. The global and regional Crs were corrected to their corresponding global or regional AOP.

**Fig. 4:**
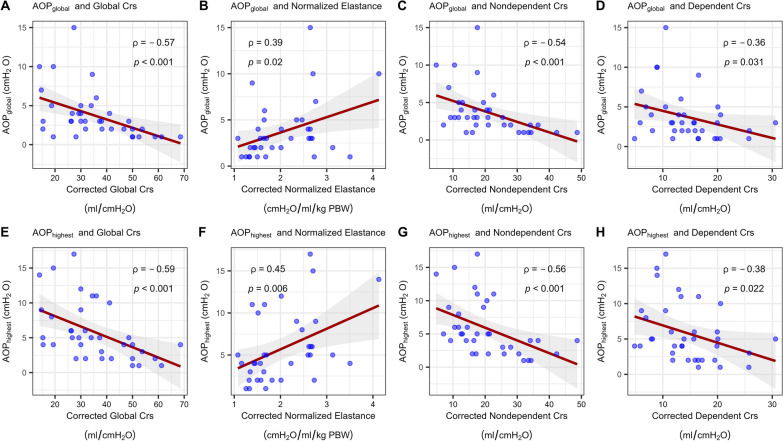
Correlation between AOP and Crs and normalized elastance corrected for corresponding AOP. AOP_global_ or AOP_highest_ and global Crs (Panel **A**, **E**), normalized elastance (Panel **B**, **F**), non-dependent Crs (Panel **C**, **G**), and dependent Crs (Panel **D**, **H**). The dark-red line represents the fitted linear regression line and the light grey bands represent the 95% confidence interval bands

There was a stronger negative correlation between AOP_global_ and the corrected Crs of global (Spearman's ρ = −0.57, *P* < 0.001) and non-dependent ventilated lung (ρ = −0.54, *P* < 0.05) than dependent ventilated lung (ρ = −0.36, *P* < 0.05). AOP_highest_ was positively correlated with global normalized elastance (ρ = 0.45, *P* = 0.006), better than for AOP_global_ (ρ = 0.39, *P* = 0.020) (Fig. 4).

### Change in slope of the low-flow pressure–time curve

When focusing on patterns of the quasi-static PT slope change, we identified 7 (19%) patients with no change, 17 (47%) with a progressive change, and 12 (33%) with an abrupt change. The BMI, P_a_O_2_/FiO_2_, and ARDS status were similar across the three patterns (Table 1). Patients exhibiting a ‘no change’ pattern were more frequently male and taller compared to the others (*P* = 0.038 and *P* = 0.017 respectively). Sixteen out of 32 ARDS (50%, including all severe ARDS patients) and 4 out of 5 patients with extrapulmonary sepsis (80%) exhibited the ‘progressive change’ pattern.

**Fig. 5:**
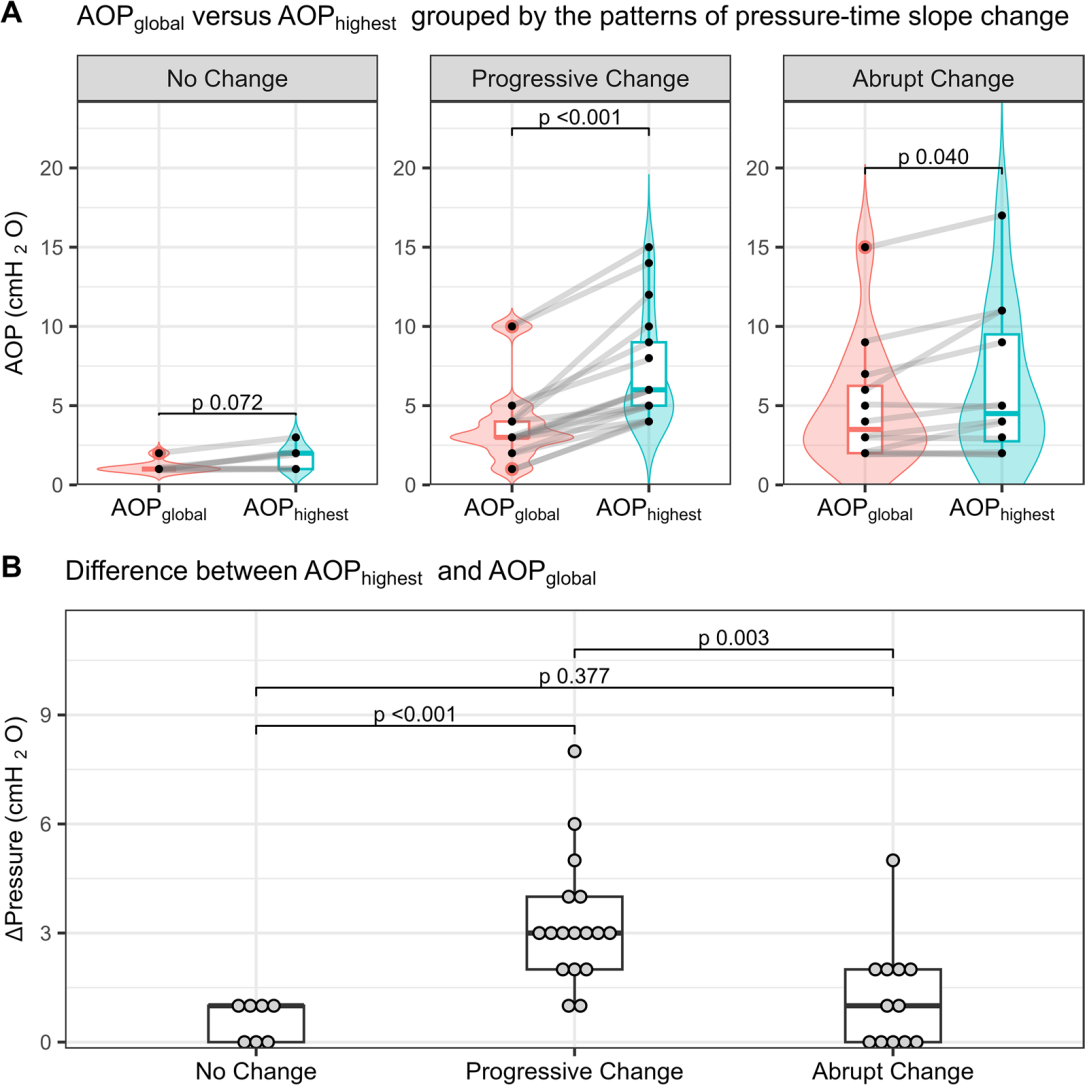
Global and highest quadrantal AOPs according to pressure–time slope change patterns during low-flow insufflation maneuver. Panel **A** shows the comparison between AOP_global_ (red) vs. AOP_highest_ (green). Panel **B** shows the comparison of the differences between AOP_highest_ and AOP_global_

In patients with a ‘progressive change’, AOP_highest_ significantly exceeded AOP_global_ (6.0 [5.0–9.0] *vs*. 3.0 [3.0–4.0] cmH_2_O, *P* < 0.001), showing a larger difference between AOP_highest_ and AOP_global_ (3.0 [2.0–4.0] cmH_2_O) than in other patterns (1.0 [0–1.0] cmH_2_O in ‘no change’ pattern, *P* < 0.001 and 1.0 [0–2.0] cmH_2_O in ‘abrupt change’ pattern, *P* = 0.003) (Fig. 5). Most patients (15, 88%) with this pattern had 1 to 3 quadrants showing regional AOPs surpassing AOP_global_ by 2 cmH_2_O or more (Additional file 1: Table S4), and 13 (76%) patients had a clinically relevant regional airway closure (any quadrantal AOP ≥ 5 cmH_2_O) (Additional file 1: Table S2). Patients with a ‘progressive change’ pattern also had a higher clinical PEEP setting at enrollment and worse Crs when compared to patients with a ‘no change’ pattern (12 [10–12] *vs*. 8 [5–9] cmH_2_O, *P* = 0.001 and 30.0 [19.4–37.3] *vs*. 52.5 [50.0–56.2] ml/cmH_2_O, *P* = 0.007; respectively) (Table 1).

### Subgroup analyses

#### ARDS

When the analysis was restricted to the 32 patients with ARDS, results were similar: 8 (25%) had AOP_global_ ≥ 5 cmH_2_O while 16 (50%) exhibited AOP_highest_ ≥ 5 cmH_2_O. AOP_highest_ was higher than AOP_global_ (4.5 [2.8–9.0] *vs.* 3.0 [2.0–4.3] cmH_2_O, *P* < 0.001) and AOP was higher in the dependent compared to non-dependent ventilated lung (4.0 [2.0–7.0] *vs*. 3.0 [2.0–5.0] cmH_2_O, *P* < 0.001) (Fig. 3B, F).

#### Obesity

Of the 11 obese patients with 10 (91%) diagnosed with ARDS, 5 (45%) patients had AOP_global_ ≥ 5 cmH_2_O and 8 (73%) had AOP_highest_ ≥ 5 cmH_2_O. AOP_highest_ exceeded AOP_global_ (8.0 [4.5–11] *vs.* 4.0 [2.5–6.5] cmH_2_O, *P* = 0.008) and AOP was also higher in the dependent ventilated lung versus non-dependent (6.0 [4.0–8.0] *vs*. 4.0 [2.5–6.5] cmH_2_O, *P* = 0.013) (Fig. 3C, G).

#### Asymmetrical AHRF

In the 6 (17%) patients presenting with asymmetrical AHRF, AOP_highest_ was higher than AOP_global_ (7.0 [6.0–9.5] *vs.* 4.0 [3.3–4.8] cmH_2_O, *P* = 0.034) but AOP of the more injured lung was only slightly and not statistically higher than the less injured lung (6.0 [3.5–9.3] *vs.* 3.5 [3.0–4.8] cmH_2_O, *P* = 0.098) (Fig. 3D, H).

## Discussion

In this prospective physiological study, we detected regional airway closure using EIT-derived quasi-static PI curves by measuring regional AOPs of different lung regions in mechanically ventilated patients with AHRF. The main findings can be summarized as follows: 1) there is a frequent heterogeneous, gravity-dependent distribution in AOPs within the lung; 2) the AOP_global_ is not a mean value but underestimates the AOP_highest_ and AOP of dependent ventilated lung. This underscores the potential of regional assessment for accurately measuring mechanics and optimizing ventilation settings; 3) the pressure–time curve analysis during low-flow insufflation maneuvers reveals that a progressive change, contrasting with no change or an abrupt change, suggests a higher likelihood of presenting different levels of regional AOP in the lung.

### Airway closure and its incidence

Airway closure can be detected at the bedside by performing a quasi-static PV or PT curve to measure AOP. Recently, Haudebourg et al. [11] described a new method that does not require a low-flow insufflation to detect an AOP. Although these methods are efficient in detecting a complete airway closure and measuring the global AOP, they ignore the non-uniformity of AOP within the lung and can underestimate the highest AOP among different lung regions, which may have implications for PEEP titration. In our study, the prevalence of clinically relevant complete airway closure (defined as AOP_global_ ≥ 5 cmH_2_O) was 25% of the patients included, a slightly lower incidence than previously reported [3, 12]. However, we observed that the prevalence of patients presenting regional airway closure with any quadrantal AOP ≥ 5 cmH_2_O was more than twice the number of patients with complete airway closure (53% versus 25%). Of note, 10 (28%) patients had no visible global airway closure but presented different levels of regional airway closure in 1 to 3 quadrants. This suggests that although complete airway closure affects a significant proportion of patients, regional airway closure is more prevalent and inhomogeneous.

The anatomical location of airway closure is debated and could be diverse across patients. Some findings in critically ill patients argued for a proximal closure (i.e. the trachea or the first bronchial generations). Malatesta et al. [13] observed airway closure of the basal segmental bronchi (i.e. the third-level bronchus) of the left lower lobe using fiberoptic bronchoscopy. By contrast, in some patients, we found airway closure only involving one quadrant of the lung while the rest of the lung was not affected, suggesting a relatively more distal rather than a very proximal location of the closure. Broche et al. [14] demonstrated involvement of small airways (18th generation) in rabbit's lung using synchrotron phase-contrast imaging. These peripheral airways lack cartilage to support their structure, making them more susceptible to collapse [15].

### Regional differences in AOP and locations

The AOP of dependent regions was consistently higher than that of non-dependent regions, which might be explained because the dependent lung is more affected by the lung weight, cardiac mass, and the cephalic displacement of the diaphragm. These observations underscore the complexity and heterogeneous nature of lung injury in ARDS, corroborating the findings of Scaramuzzo and colleagues [16]. In the dependent region it is more difficult to distinguish between airway collapse or simply lung reaeration.

A correlation exists showing that lower respiratory compliance is associated with higher AOP. Moreover, the stronger positive correlation between the AOP_highest_ and normalized elastance compared to global AOP is in line with results of Rozé et al. [5] and Bastia et al. [17] in asymmetrical ARDS: the more the region of the lung is injured, the higher the local AOP. These are also coincident with the findings of Hinz J et al. that in patients with acute respiratory failure, the regional lower inflection points (LIPs)—which show the airway pressure to keep all lung regions open—obtained from the regional PV curves measured by EIT progressively increased from ventral to dorsal regions, and the regional LIPs underestimated the global measurements [10].

### Shape of the pressure–time curve

Identifying a global AOP is straightforward when an abrupt change in the slope of the quasi-static PT curve is observed. Our study found that nearly half of the ventilated AHRF patients, including all severe ARDS patients, displayed a progressive change in the slope of the PT curve. This more subtle shift can make precise bedside determination of global AOP more challenging, potentially leading to misclassification. A progressive change in slope of PT curve seems to indicate a gradual alteration in regional AOPs and lung compliance, possibly reflecting a dynamic process of airway opening and lung recruitment. In our analysis, a large majority (15 out of 17) of patients with this pattern had 1 to 3 quadrants, mainly in the dependent lung, showing regional AOPs surpassing the global AOP by 2 cmH_2_O or more. Furthermore, more than half of these patients (9 out of 17) had regional airway closure without concurrent global or complete airway closure. This underlines a pronounced heterogeneity in airway closure and lung injury across different lung regions when a progressive pattern is observed.

### Clinical implications

The global AOP derived from a low-flow PV curve potentially serves as a logical minimal PEEP level setting, especially in cases marked by an abrupt slope change in slope. However, in scenarios exhibiting a progressive slope change pattern, relying solely on global AOP for PEEP setting may not be sufficient. First, the set PEEP does not reflect the real regional alveolar pressure inducing errors in the calculation of the driving pressure, the most important variable to predict the risk of mortality [18]. Coudroy et al. [19] demonstrated that the correction for airway closure led to a reduction in driving pressure (mean difference, − 0.9 cmH_2_O) and in the elastance of the respiratory system. Recent data suggested that a low PEEP set to minimize overdistention was not safe in a highly recruitable ARDS model, and underestimating PEEP can result in deleterious consequences [20]. Airway closure might also be the cause of lesions in the airways, due to repeated opening and closing of the bronchi at each respiratory cycle. Last, airway closure can quickly provoke denitrogenation atelectasis and further lung collapse, worsening both gas exchange and injury to the lung tissue remaining aerated [21].

We believe that the consideration of regional airway closure should be a prerequisite for personalized ventilator settings. This is especially true in obese and ARDS patients in whom the prevalence of this phenomenon is high in our series and in the literature [19, 22, 23]. A PT curve during a low-flow insufflation maneuver is readily accessible on every ICU ventilator, and our routine use shows it is a safe and well-tolerated procedure. Patients with a progressive slope change observed from the PT curve should be suspected of having an uneven regional AOP distribution. In this case, the approach to setting PEEP to prevent regional atelectasis and overdistension for lung-protective ventilation strategies suggests the importance of precise regional AOP assessment. Some patients might exhibit very high regional AOP (maximum of 8 cmH_2_O over global AOP in our cohort) not reflected by global values. In the vast majority (89%) of patients in our study, the clinical PEEP level was generally set higher than both global and highest regional AOP, especially in patients with a progressive slope change pattern, who received higher PEEP levels.

### Limitations

While our study provides valuable insights into AOP heterogeneity and its correlation with slope change patterns, several limitations should be acknowledged including the limited number of patients and lack of intervention to adjust the PEEP. We did not assess the impact of regional AOP heterogeneity, slope change patterns of PT curve, and the consequences of setting PEEP below the global or highest regional AOP. We assessed AOP in supine (mostly semi-recumbent) positions and our results cannot be generalized to other body positions. For instance, lateral or prone positions might affect airway closure differently due to direct bronchial compression by the mediastinum and an increase in intra-abdominal pressure [24, 25]. The spatial resolution of EIT in the cranio-caudal direction provides a slice thickness of up to 10 cm (5 cm on either side of the EIT belt) [26]. This limited cranio-caudal exploration could potentially overlook variations in AOPs in the unmonitored upper lobes [27]. Further research is warranted to explore their potential implications and guide clinical strategies based on assessing PEEP settings according to the global or highest quadrantal AOPs, especially in patients with progressive slope change patterns of PT curve.

## Conclusion

Our study highlights the significant heterogeneity and gravity-dependent distribution of regional AOPs in patients with AHRF/ARDS on mechanical ventilation. We observed that the global AOP does not represent a mean value but may underestimate the highest regional AOP; a progressive airway pressure slope change during the low flow inflation indicates the presence of several and higher regional AOPs. Analysis of slope change patterns in the pressure–time curve provides easy and valuable clinical insights into regional AOP variations and lung mechanics, helping with personalized ventilation strategies tailored to the individual patient's lung condition.

## Supplementary Information

Additional file 1.

## Data Availability

The data used in the present study are available from the corresponding author upon reasonable request.

## Abbreviations

AHRF: Acute hypoxemic respiratory failure
Paw: Airway pressure
AOP: Airway opening pressure
PV: Pressure–volume
PT: Pressure–time
PEEP: Positive end-expiratory pressure
ARDS: Acute respiratory distress syndrome
EIT: Electrical impedance tomography
SMH: St. Michael’s Hospital
APUH: Amiens-Picardie University Hospital
ICU: Intensive care unit
PB: Puritan Bennett
FiO_2_: Fraction of inspired oxygen
RR: Respiratory rate
AOP_vent_: AOP measured from the quasi-static PV curve
ROI: Region of interest
ROI_UR_: Upper right region of interest
ROI_UL_: Upper left region of interest
ROI_LR_: Lower right region of interest
ROI_LL_: Lower left region of interest
AOP_global_: Global AOP measured by EIT
AOP_highest_: Highest quadrantal AOP
Crs: Respiratory system compliance
BMI: Body mass index
P_a_O_2_: Partial pressure of arterial oxygen
APACHE II: Acute physiology and chronic health evaluation II score
SOFA: Sepsis-related Organ Failure Assessment score
PBW: Predicted body weight
FEV1: Forced expiratory volume in the first second
GOLD: Global initiative for chronic obstructive lung disease
ROI_Dep_: Dependent lung region of interest
ROI_Left_: Left lung region of interest
ROI_Right_: Right lung region of interest
