# Supplementary material for: Distribution of airway pressure opening in the lungs measured with electrical impedance tomography (POET): a prospective physiological study
Source: Crit Care. 2025 Jan 16;29:28. doi: 10.1186/s13054-025-05264-3 (PMC11740639; doi:10.1186/s13054-025-05264-3)

# Distribution of airway pressure opening in the lungs measured with electrical impedance tomography (POET): a prospective physiological study

# Supplemental Material

Sample size determination and post-hoc power analysis

In this physiological study, we calculated our sample size based on the desired statistical power to achieve our primary outcome with high precision. We aimed to enroll 30 patients assuming a 30% incidence of complete airway closure in patients under mechanical ventilation, an anticipated 75% incidence of regional airway closure in our study group, a confidence level of 80%, and a precision level of 10%. We reassessed the sample size after the inclusion of the first 15 patients because we observed a lower incidence of global and regional airway closure than expected. We also noticed technical issues during the study procedure in some patients (such as gas-trapping or inadequate EIT signals), as initially observed at St. Michael’s Hospital, Unity Health, Toronto, Canada (see Fig 1). Consequently, we decided to enlarge the sample size to 46 patients. Both original and amended sample sizes were approved by our Research Ethics Board. Ultimately, we analyzed data from 36 patients.

Our post-hoc power analysis indicated that we achieved a strong statistical power (1.0) for the Wilcoxon signed-rank test (matched pairs) with a sample size of 36, effect size of 1.0, and alpha level of 0.05 when comparing the global AOP and the highest regional AOP by quadrant. Furthermore, we also attained a sufficient power (0.9) for the Chi-square test (sample size = 36, ratio 2.12, alpha level = 0.05) when comparing the prevalence of complete airway closure and regional airway closure. Sample size analyses were conducted in G*Power 3.1.9.4.

Methodological aspects

***The standard operating procedure of the low-flow insufflation maneuver in POET study***

As for the low-flow insufflation maneuver, we initially tested the airway closure with Evita XL by one ‘low flow PV-loop’ maneuver with clinical PEEP and respiratory rate, but some cases with gas-trapping or auto-PEEP before the maneuver were found which impacted the AOP measurements (see flowchart). With Servo-i and PB840 we performed the maneuver with synchronized intermittent mandatory ventilation (SIMV) mode as the online posted procedure (<https://crec.coemv.ca/ri-ratio>), but we found that some cases had gas-trapping before the first breath identified by flow tracing or the global impedance tracing which couldn’t drop to the minimal lever before the first breath compared to the second.

To limit auto-PEEP, it would be helpful to use the time constant for each patient because 3-4 time constants of exhalation time were needed to ensure emptying and prevent auto-PEEP. Practically, it’s challenging to manage the exhalation time before the maneuver immediately after decreasing clinical PEEP to zero PEEP with a large end-expiratory lung volume at clinical PEEP to exhale. So we updated the maneuver protocol as follows: using Evita XL, we dropped the clinical PEEP to zero or 5 cmH2O and reduced the respiratory rate to 5 for 2 breaths before initiating the maneuver. With Servo-i and PB840, we recorded 2 low-flow insufflation maneuvers with zero PEEP after dropping the respiratory rate to 5/min or 3/min, and only the second maneuver was kept for analysis. These strategies worked well.

***Synchronization of EIT data and Biopac data***

In cases where PressurePod was unavailable, a special cable was used to connect Evita XL ventilator to PulmoVista 500, facilitating collection of gas flow, volume, and airway pressure data. EIT impedance and pressure signals were synchronized with Paw and flow measurements from Biopac resampled to 50 Hz, rectifying a 0.232-second delay (offered by Dräger) on Evita XL to impedance signal on PulmoVista 500, alternatively addressing overlapping the two Paw tracings from Biopac or Fluxmed to PressurePod on PulmoVista 500.

***The regions of interest (ROIs) modification of EIT images in POET study***

For the ROIs modification of EIT images, we determined the ventilated contour (Fig S1) as the upper and lower boundaries of the functional lung. This ventilated contour was obtained from the maximal ventilation of the lung during the whole recording data with baseline ventilation on clinical PEEP and the low-flow insufflation maneuver. We didn’t perform a higher PEEP than clinical PEEP to assess recruitability and recruit the most dependent alveoli. We also limited the maximal airway pressure during the maneuver to the plateau pressure (lower than 30 to 40 cmH_2_O) on clinical ventilator settings. In our study, most patients' global and highest regional AOPs were lower than the clinical PEEP levels. Therefore this ventilated contour strategy was sufficient for AOP analysis which focused on the initial insufflation. Because of this analysis strategy, the ventilation boundaries varied from patient to patient, mainly in the dependent and non-dependent regions.

***Definition of global and regional AOPs detected by EIT***

We initially tried converting the 15 ml volume threshold defined by AOP_vent_ into the global change in impedance (∆Z) threshold by the ratio of the global maximal ∆Z to the inflated volume at the end of insufflation, then using the distribution percentages in this insufflation multiplied this global ∆Z threshold as the regional threshold to define regional AOP. However, this method did not fit well in some patients potentially due to the accuracy of volume data from different ventilators and different regional compliance (or time constants). Please make a note of the following information: the success of this method depends on the ratio of global maximal ∆Z to the inflated volume at the end of insufflation. Before data collection, we calibrated the Biopac flow and performed an airtight check on Evita XL using an occluded circuit connected to a filter applied with the pneumotachograph. However, we encountered significant variations in both flow and volume. It's worth noting that data from the Biopac system is more accurate and reliable, while data from the ventilator may be inconsistent due to an unstable flow sensor.

Additionally, we observed the impedance signal was somewhat filtered out by the low-pass filter (with a cut-off frequency of 50/min), particularly during the final phase of insufflation maneuver in some patients (Fig S3). So we chose to use the unfiltered impedance signal for the final AOP analysis. Therefore, we defined the global or regional AOP detected by EIT as the pressure at which the insufflation impedance exceeded the baseline limit, which was determined as cardiac-induced noise, calculated as the mean plus 2 standard deviations of the impedance signal. This baseline duration included at least one complete heartbeat cycle period to estimate variation induced by heart-beating best and with minimal influence of ventilation with almost zero flow at end-expiration before the maneuver (Fig S4).

In conclusion, our approach enabled the measurement of AOPs in regions with significant variations in ventilation percentages, without relying on precision instruments such as Biopac or Fluxmed systems as long as the pressure signal synchronized to the impedance signal with a proper low-flow insufflation maneuver. Because of the relatively low sample rate of EIT (50 Hz) for low-flow measurement, we rounded up each AOP value to an integer. Our methodology of regional AOPs is potentially actionable for intensivists or respiratory therapists in clinical practice.

Table S1. Prevalence of clinically relevant airway closure in the 36 patients included.

| Clinically significant airway closure | n (%) |
| --- | --- |
| 36 patients included |  |
| AOP_global_ ≥ 5 cmH_2_O | 9 (25) |
| AOP_highest_ ≥ 5 cmH_2_O | 19 (53) |
| AOP_highest_ ≥ 5 cmH_2_O & AOP_global_ ≥ 5 cmH_2_O | 9 (47) |
| AOP_highest_ ≥ 5 cmH_2_O & AOP_global_ < 5 cmH_2_O | 10 (53) |
| 144 quadrants in 36 patients’ lungs |  |
| AOP of any quadrantal lung ≥ 5 cmH_2_O | 52 (36) |
| AOP of upper right lung ≥ 5 cmH_2_O | 10 (19) |
| AOP of upper left lung ≥ 5 cmH_2_O | 14 (27) |
| AOP of lower right lung ≥ 5 cmH_2_O | 13 (25) |
| AOP of lower left lung ≥ 5 cmH_2_O | 15 (29) |

Table S2. Locations of clinically relevant regional airway closure in 19 patients with AOP_highest_ ≥ 5 cmH_2_O.

| Locations of regional AOP ≥ 5 cmH_2_O | | Patients  n (%) | Slope change pattern of PT curve | |
| --- | --- | --- | --- | --- |
|  |  |  | Progressive change  n | Abrupt change  n |
| AOP_global_ < 5 cmH_2_O | ROI_UL_ | 2 (11) | 2 | 0 |
|  | ROI_LR_ | 1 (5) | 1 | 0 |
|  | ROI_LL_ | 2 (11) | 2 | 0 |
|  | ROI_Left_ (ROI_UL_ + ROI_LL_) | 2 (11) | 1 | 1 |
|  | ROI_Right_ (ROI_UR_ + ROI_LR_) | 1 (5) | 1 | 0 |
|  | ROI_Dep_ (ROI_LR_ + ROI_LL_) | 1 (5) | 1 | 0 |
|  | ROI_UL_ + ROI_LR_ + ROI_LL_ | 1 (5) | 1 | 0 |
| AOP_global_ ≥ 5 cmH_2_O | ROI_UR_ + ROI_UL_ + ROI_LR_ + ROI_LL_ | 9 (47) | 4 | 5 |
|  | Total | 19 | 13 | 6 |

Table S3. Distribution of high regional airway opening pressure categorized according to difference between AOP_highest_ and AOP_global_. The difference between AOP_highest_ and AOP_global_ ranged from 0 to 8 cmH_2_O. Twenty (56%) patients had this difference of AOP ≥ 2 cmH_2_O and were defined as high regional AOP. Some patients had multiple quadrants with high regional AOP.

| Difference between  AOP_highest_ and AOP_global_  (cmH_2_O) | | Patients  n (%) | Quadrants of ROI | | | | |
| --- | --- | --- | --- | --- | --- | --- | --- |
|  |  |  | ROI_UR_  n | ROI_UL_  n | ROI_LR_  n | ROI_LL_  n | Total  n |
| 0~1 | | 16 | - | - | - | - | - |
| ≥2 | 2~3 | 14 (70) | 1 | 5 | 5 | 13 | 24 |
|  | 4~5 | 4 (20) | 0 | 4 | 0 | 2 | 6 |
|  | 6~7 | 1 (5) | 0 | 0 | 0 | 1 | 1 |
|  | 8 | 1 (5) | 0 | 0 | 1 | 0 | 1 |
|  | Total | 20 | 1 | 9 | 6 | 16 | 32 |
| Total | | 36 |  |  |  |  |  |

Table S4. Locations of high regional AOP in 20 patients with differences between AOP_highest_ and AOP_global_ ≥ 2 cmH_2_O.

| Locations of  high regional AOP | Patients  n (%) | Slope change pattern of PT curve | |
| --- | --- | --- | --- |
|  |  | Progressive change  n | Abrupt change  n |
| ROI_UL_ | 2 (10) | 1 | 1 |
| ROI_LR_ | 1 (5) | 1 | 0 |
| ROI_LL_ | 7 (35) | 6 | 1 |
| ROI_Left_ (ROI_UL_ + ROI_LL_) | 5 (25) | 4 | 1 |
| ROI_Right_ (ROI_UR_ + ROI_LR_) | 1 (5) | 0 | 1 |
| ROI_Dep_ (ROI_LR_ + ROI_LL_) | 2 (10) | 1 | 1 |
| ROI_UL_ + ROI_LR_ + ROI_LL_ | 2 (10) | 2 | 0 |
| Total | 20 | 15 | 5 |

Fig S1. Regions of interest defined from electrical impedance tomography images.

A cross-section image of the thorax was acquired from the Pulmovista between the 4^th^ and 5^th^ intercostal space. Impedance change defined by color-coding scheme: impedance increase is represented as blue-white, while impedance decrease is represented as purple. The light grey line represents the thoracic contour. The white dotted line represents the regions of the lung where ventilation-related impedance changes occur (ventilated contour). The center of the ventilated lung (red circle) was defined based on the ventilated contour's maximal height and the central axis of the thoracic contour. Electrical impedance tomography (EIT) image was divided into several regions of interest (ROI) representing the following lung area: upper right (ROI_UR_), upper left (ROI_UL_), lower right (ROI_LR_), lower left (ROI_LL_), non-dependent (ROI_NON-DEP_), dependent (ROI_DEP_), right (ROI_RIGHT_), and left (ROI_LEFT_) lungs.

Fig S2. Bland-Altman plot comparing global airway opening pressure from quasi-static pressure-impedance curve and pressure-volume curve.

The median AOP_global_ (from quasi-static pressure-impedance curve) and AOP_vent_ (from quasi-static PV curve) were 3.0 [2.0-4.3] cmH_2_O and 3.0 [1.5-4.0] cmH_2_O respectively (*P* = 0.201). Bland-Altman analyses showed a good agreement between the two methods. The solid line indicates the mean ratio of AOP_global_ to AOP_vent_. The dotted lines indicate the limits of agreement.

Fig S3. The impedance signal was somewhat filtered out by the low-pass filter on EIT.

Panel A is the PV curves obtained from the patient (patient #24) (in blue) and measured in the bench model with an occluded ventilator circuit (in red). Panel B is the global PI curves obtained from the same patient with and without the low-pass filter (a cut-off frequency of 50/min) in dark blue and green respectively. At the last phase of the low-flow insufflation maneuver, the rectangular shaded areas in light red are the Paw from 25 to 26.6 (end of the maneuver) cmH_2_O. The global impedance with the low-pass filter did not increase as the volume increased at the last phase of the maneuver.


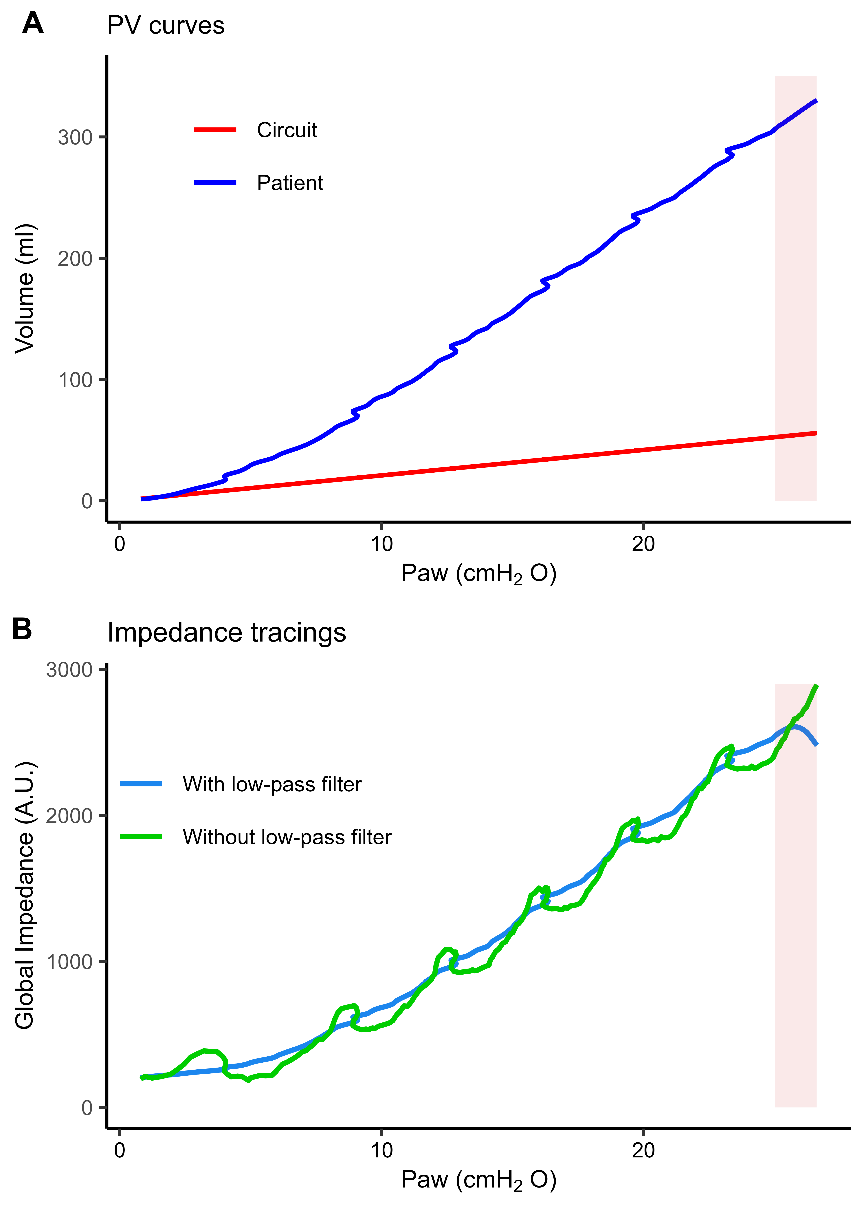


Fig S4. Examples of AOP measurement detected by EIT.

Panel A, B, and C are tracings of patient #33 with an abrupt slope change of PT curve. Panel D, E, and F are tracings of patient #16 with a progressive slope change of PT curve. Panel A and D are the global impedance-time tracings with and without low-pass filter, the red lines are the upper limit calculated as the mean plus 2 standard deviations of the baseline impedance (in purple rectangular shaded areas). During the insufflation maneuver (in yellow rectangular shaded areas), when the impedance increases above this baseline limit (shown as the black arrow pointed, 4.12sec for patient #33 and 4.74sec for patient #16), the airway opens and inflation starts. Panel B and E are the pressure-time tracings, and black arrows show the AOPs: 3.4 cmH_2_O rounded up to 4 cmH_2_O for patient #33 and 9.6 cmH_2_O rounded up to 10 cmH_2_O for patient #16. Panel C and F are the flow-time tracings with relatively zero flow for 1-second duration including one complete heartbeat cycle period at end-expiration before the maneuver (in purple rectangular shaded areas).


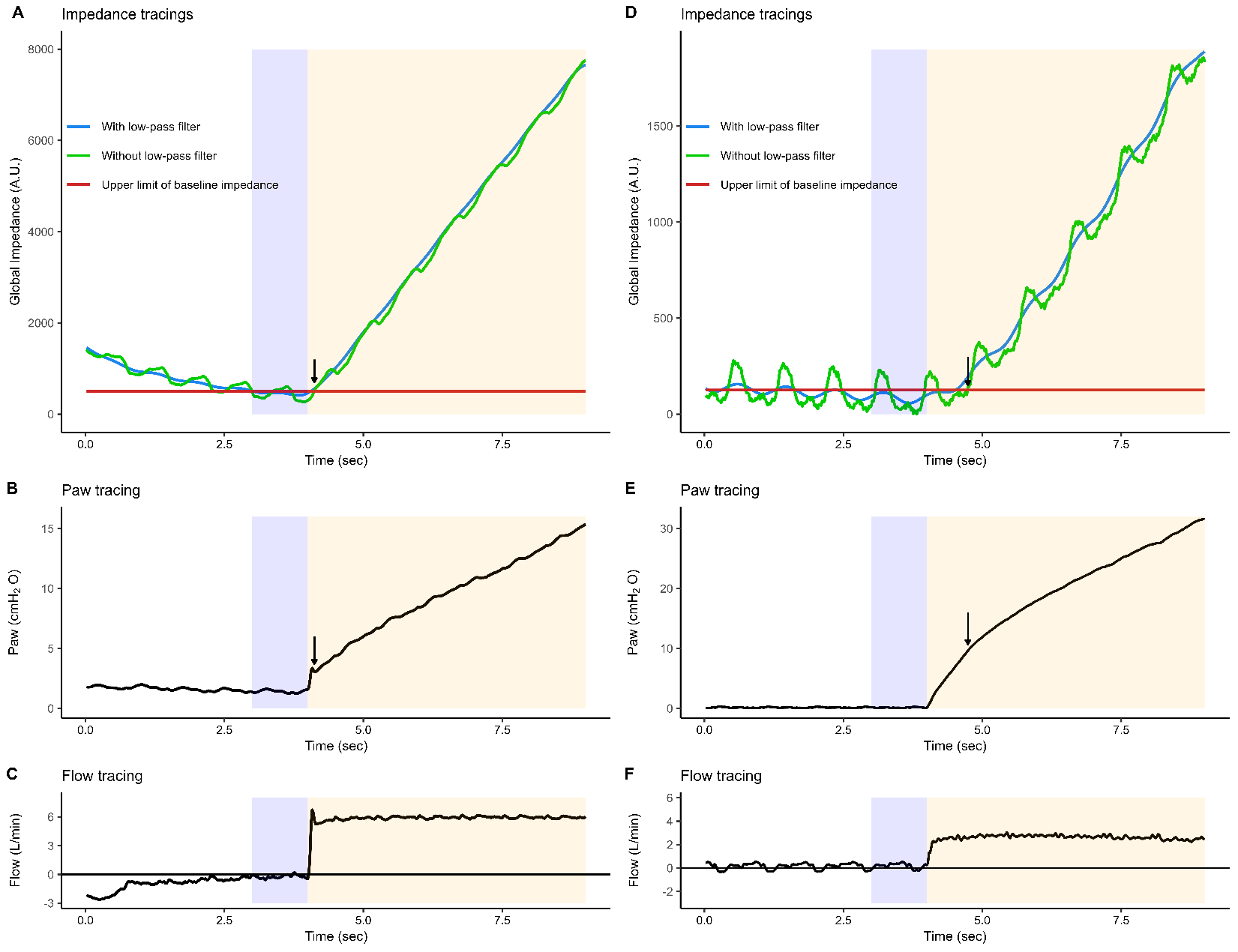

Supplement: Supplementary file 1 — Additional file 1. [file 13054_2025_5264_MOESM1_ESM.docx]
